# Localization of C-Fos-Induced Growth Factor (*Figf*) mRNA Expression in the Mouse Uterus during Implantation

**DOI:** 10.4172/2161-038X.S1-003

**Published:** 2012-01-25

**Authors:** Charles A Scott, Kirsten S. Eckstrum, Brent M. Bany

**Affiliations:** 1 Departments of Physiology, Southern Illinois University School of Medicine, Carbondale, Illinois, USA; 2 Departments of Obstetrics & Gynecology, Southern Illinois University School of Medicine, Carbondale, Illinois, USA

**Keywords:** Uterus, Decidualization, Endometrium, c-FOS-induced growth factor

## Abstract

The purpose of this study was to characterize the localization of *Figf* mRNA in the mouse uterus during embryo implantation. Strong *Figf* mRNA hybridization signals were seen in the primary decidual zone just after the onset of implantation from Days 4.5–6.5. On Day 7.5, this expression continued around the concept us, but in addition we observed high expression of *Figf* mRNA in the endothelial cells that line the forming vascular sinusoids in the lateral me some trial decidua. Interestingly, on Days 8.5 this high expression continued in the endothelial cells of sinusoids in the lateral me some trial decidual tissue but not in the decidual cells surrounding the concept us. As implantation and placental development finished, *Figf* mRNA expression remained in the endothelial cells of the sinusoids and spiral arterioles of the decidua basalis. Interestingly, *Flt4* mRNA was localized to the endothelial cells lining the sinusoids that form during implantation. Since the endothelial cells of the me some trial sinusoids exhibit a high level of proliferation, we speculate that FIGF-FLT4 signaling may play a role in their formation and function during implantation. This work will provide a basis for further research on the potential role of FIGF-FLT4 signaling in endometrial angiogenesis during implantation in mice.

## Introduction

Implantation of the concept us begins with the attachment of the concept us to the uterine wall and ends with the formation of the placenta [1]. During this time the uterus must provide the proper environment to support placental and embryo development. In rodents and humans, implantation involves the transformation of the endometrial stroma into decidual tissue which is commonly referred to as decidualization [2,3]. During decidualization there is a rapid proliferation of endometrial stromal fibroblast cells, which is followed by their transdifferentiation into epithelioid-like decidual cells. As a consequence, there is a dramatic increase in endometrial tissue mass during decidualization in the pregnant uterus. Dramatic changes in the uterine vasculature accompany and support the process: Angiogenesis and vascular remodeling in the endometrium are key features of implantation [4].

The vascular endothelial growth factor (VEGF) family and associated receptors play key roles in angiogenesis or lymphangiogenesis and has been the subject of many reviews [5–8]. Briefly, Vegfa, Vegfb, Vegfc, *Figf* and Plgf genes are all members of the VEGF family of ligands. The genes that encode receptors to which one or more of the VEGF family of ligands bind are called FMS-like tyrosine kinase 1 (Flt1), kinase insert domain receptor (Kdr) and FMS-like tyrosine kinase 4 (*Flt4*). VEGFA binds to and activates FLT1 and KDR. VEGFB and PLGF bind to and activate FLT1. VEGFC binds to and activates KDR and *FLT4*. Finally, human *FIGF* binds and activates both KDR and *FLT4* but the mouse protein only does so for *FLT4* [9–12]. In general, FLT1 and KDR ligands are considered the major regulators of angiogenesis in the adult while *FLT4* ligands play mainly a role in lymphangiogenesis in the adult [5].

VEGF family members which bind to the angiogenic VEGF receptors are believed to play a key role in endometrial angiogenesis during implantation in mice. Initial studies showed Flt1 plus Kdr expression, as well as VEGFA-binding, occurs in a subset of endothelial cells in the mouse endometrium in areas undergoing decidualization, while Vegfa expression occurs in decidual cells and some endothelial cells [9,13]. Subsequently, Vegfa expression by uterine natural killer (uNK) cells and trophoblast giant cells were demonstrated, raising them as potential sources of the endometrial VEGFA that plays a role in uterine angiogenesis during implantation [14,15]. The major source of Plgf expression in the mouse uterus during implantation is the trophoblast giant cells [14] and by mid-pregnancy uNK cells also become a major source [16]. These observations suggest that VEGFA and PLGF play key roles in mediating the angiogenic changes in the endometrial vasculature during implantation in mice.

Although *FLT4* is thought to play a role in lymphangiogenesis in adults, several recent observations suggest that *FLT4* and its ligand *FIGF* may play a role in endometrial angiogenesis during implantation in mice. First, lymphatic vessel endothelial hyaluronan receptor 1 (LYVE1)-positive lymphatic vessel endothelial cells are predominantly found in the myometrial connective tissue between the muscle layers of the myometrium during implantation with very little endometrial presence at the endometrial-myometrial border [17]. Second, *FLT4* protein is localized in the primary decidual zone to platelet/endothelial cell adhesion molecule 1 (PECAM1)-positive endothelial cells, during early and later stages of implantation [18]. Third, *FIGF* causes the enlargement and proliferation of mouse endometrial, but not myometrial, blood vessel endothelial cells [17]. Finally, blockage of *FLT4* activity significantly reduces decidual blood vessel numbers but does not prevent successful pregnancy [18]. These observations suggest that a function of *Figf* expression in the mouse uterus during implantation is to modulate angiogenesis or vascular remodeling of the endometrial vasculature.

One key aspect of the potential regulation of endometrial angiogenesis by *FIGF* presently requires clarification. To our knowledge, only very limited available data localizes *Figf* expression to the mesometrial endometrium during implantation in mice on Day 7.5 of pregnancy [19]. Therefore, the purpose of this study was to fully characterize the localization of *Figf* mRNA expression in the mouse uterus during embryo implantation and placental development in mice from Day 4.5 to 11.5 of pregnancy. Further, since two splice variants exist for *Figf* mRNA [20], we determined if one or both are expressed in the uterine tissue. Finally, to complement previous immunohistochemical data of *FLT4* localization [18], we also localized *Flt4* mRNA in the mouse uterus during implantation.

## Materials and Methods

### Animals

All animal work was approved by the Southern Illinois University IACUC committee. CD1 mice were purchased from Charles River Laboratories (Wilmington, MA). They were housed under controlled light conditions (lights on 6 a.m. to 8 p.m.) with free access to food and water. Pregnant animals were obtained by mating 10–12 week old females with mature males and the morning a vaginal plug was discovered was considered Day 0.5 of pregnancy. Mice were killed on Days 4.5–11.5 of pregnancy and uteri were collected.

### Tissue collection and processing

Implantation (IS) and non-implantation (NIS) segment tissues of the pregnant uteri were separated. For RNA isolation, concept uses were dissected out of IS tissue. Tissues were either collected for RNA isolation or for in situ hybridization. Tissues collected for RNA isolation were homogenized in Trizol Reagent (In Vitrogen), while those used for in situ hybridization were fixed in 4% paraformaldehyde in PBS for 24 h.

### In situ hybridization

Fixed tissue was processed into paraffin blocks using standard methods, then sectioned (5 µm) using a rotary microtome. In situ hybridization was conducted as previously described [21] using digoxigenin (DIG)-labeled riboprobes and BCIP/NBT as the colorimetric substrate. The slides were counterstained with nuclear fast red prior to mounting coverslips. The mRNA signals were purple and nuclei stained red. Photomicrographs were captured using a Leica CTR 5000 fluorescence microscope (Leica) equipped with a Retiga 2000 JR QImaging Camera and Qcapture Pro software (QImaging, Burnaby, BC).

The cDNA clones for mouse *Figf* (clone ID 30286444) and *Flt4* (clone ID 5291949) were purchased from Open Biosystems (ThermoFisher Scientific, Huntsville, AL). Plasmid DNA harboring each cDNA clone was isolated using an E.Z.N.A. Fast filter Plasmid Maxi Kit as recommended by the manufacturer (Omega BioTek, Norcross, GA). The cDNA was then used to prepare antisense and control sense riboprobes using methods described previously [22]. No signals were seen in all hybridizations where sense probes were used (data not shown).

### Reverse-transcription polymerase chain reaction (RT-PCR)

RT-PCR was used to assess the presence of each of the two *Figf* mRNA splice variants. The primers used were previously described and produce 771 and 636 bp amplicons from the alternatively spliced transcripts, called *Figf*_358_ and *Figf*_326_, which encode the 358 (VEGF_358_) and 326 (VEGF_326_) amino acid forms of *FIGF* protein, respectively [20]. RT-PCR was carried out using Improm II reverse transcriptase (Promega, Madison, WI) and TopTaq PCR master mix (Qiagen, Valencia, CA) following the procedures recommended by the manufacturers. PCR was carried out using an Eppendorf Mastercycler thermocycler (Fisher Scientific, Pittsburgh, PA) programmed for 36 cycles of 94°C melt, 58°C annealing and 72°C extension steps for 15, 45 and 20 seconds, respectively. After a final incubation for 7 minutes at 72°C the samples were subjected to agarose gel electrophoresis in the presence of ethidium bromide. The resultant gels were imaged using a UV trans-illuminator and a Kodak EDAS290 Gel Documentation system (Fisher Scientific).

### Bromodeoxyuridine (BrdU)-CD34 double immunohistochemistry

Mice were injected with BrdU (Sigma, Saint Louis, MO) at 4 h prior to tissue collection as previously described [22] in order to visualize cells undergoing proliferation. Uterine tissues were fixed, embedded into paraffin blocks then sectioned as above. After sections were de-waxed and hydrated using routine techniques, they were incubated with 3% hydrogen peroxide in 1× PBS for 10 min to block endogenous peroxidase activity. Sections were digested with 0.2% trypsin in PBS at 37°C for 10 min for antigen retrieval, then washed with PBS. Sections were incubated with 1.5M HCl for 15 min, washed with water (10 dips), incubated in borate buffer (0.1M, pH 8.5) for 10 min, and then washed twice in PBS containing 0.05% tween (PBST) for 5 minutes per wash. Subsequent incubations were carried out using antibody amplifier trays from ProHisto (Columbia, SC) and a horizontal shaker. Sections were incubated in blocking buffer (2% donkey serum in PBST) for 60 min. Sections were then incubated with sheep anti-BrdUIgG (0.3µg/ml, Biodesign International, Saco, ME), followed by two 5 minute washes in PBST. Sections were covered with 2.5µg/ml alkaline phosphatase conjugated streptavidin (Jackson ImmunoResearch Inc., West Grove, PA) in blocking medium for 30 min, then washed in PBS containing 0.6 mg/ml of tetramisole hydrochloride (Sigma) for 5 min to block endogenous phosphatase activity. Color development was then carried out using a VECTOR Blue Alkaline Phosphatase kit as recommended by the manufacturer (Vector Labs, Burlingame, CA) in the presence of 0.6 mg/ml tetramisole until a blue precipitate formed. Sections were next washed in water (10 dips), incubated twice with PBS for 10 minutes, then incubated for 60 minutes in blocking medium. After incubation with rat anti-mouse CD34 antigen (CD34) IgG (0.1 µg/ml, Cedarlane Laboratories, Hornby, ON) in blocking buffer for 1 hour, the sections were washed twice for 5 min in PBST. Sections were then incubated with 4ug/ml donkey anti-rat peroxidase-conjugated IgG (Jackson ImmunoResearch) in blocking buffer for 60 min, followed by two washes with PBS. Color development was then carried out using an ImmPact DAB Peroxidase Substrate kit as recommended by the manufacturer (Vector Laboratories) until a brown precipitate formed. After the sections were washed in water they were dehydrated and mounted with coverslips using Clearium Mounting Medium as directed by the manufacturer (Surgipath Medical Industries Inc., Richmond, IL). No signals were seen for BrdU in tissue from mice not injected with BrdU or in sections stained with purified sheep IgG (Jackson ImmunoResearch) in place of the anti-BrdUIgG (data not shown). No signals were seen for CD34 when purified rat IgG (Jackson ImmunoResearch) was used in place of the anti-CD34 IgG (data not shown).

## Results

### *Figf* mRNA localization in the uterus during implantation

**Figure 1:**
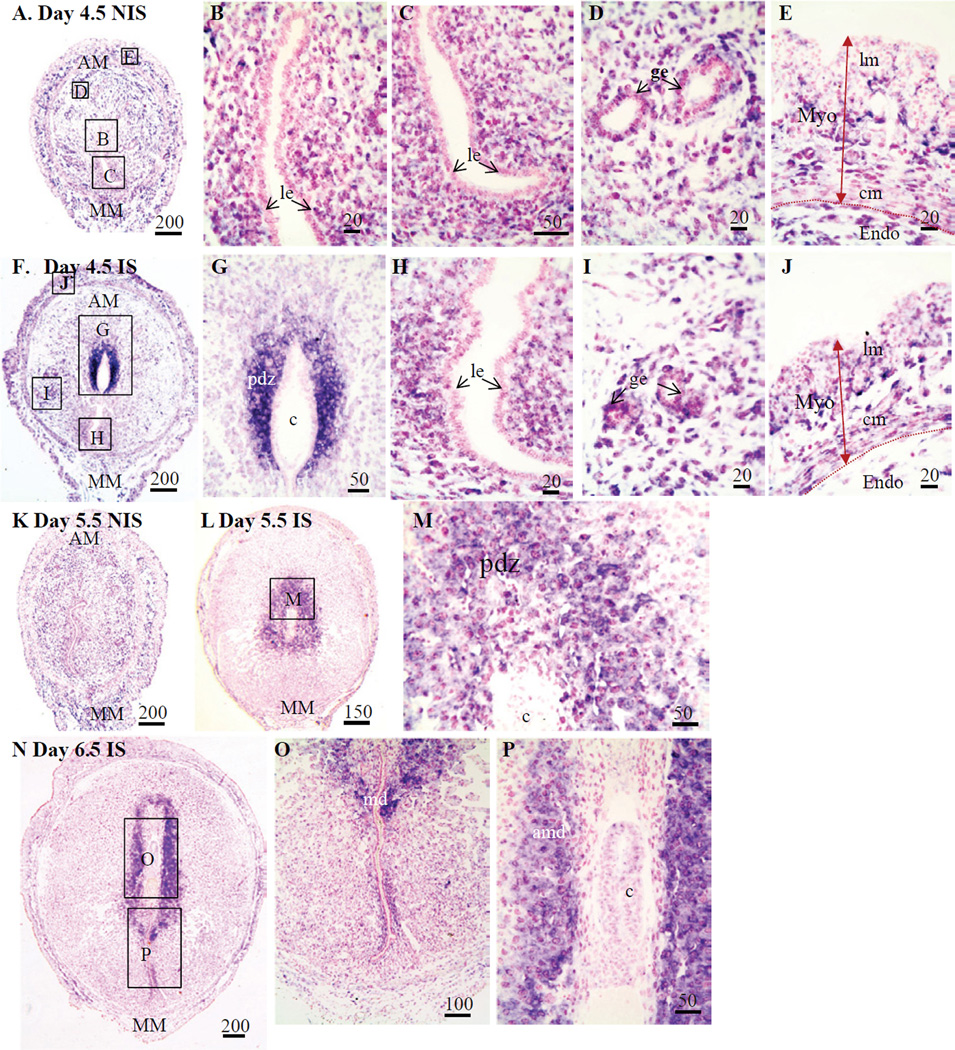
Localization of *Figf* mRNA in non-implantation (NIS) and implantation (IS) tissue segments from Day 4.5–6.5 pregnant mice. (**A–E**) Day 4.5 NIS, (**F–J**) Day 4.5 IS, (**K**) Day 5.5 NIS, (**L–M**) Day 5.5 IS and (**N**) Day 6.5 NIS and (**O–P**) Day 6.5 IS. These are representative of at least 3 independent samples. Global linear adjustments of the brightness and color level were made on the photomicrographs to more accurately represent what was seen on the slides under the microscope. Numbers above scale bars are in microns. Hybridization signal is purple while nuclear stain is pink. All sections in photomicrographs are oriented mesometrial (MM) and anti-mesometrial (AM) regions down and up, respectively. Abbrev: AM, antimesometrial; amd, antimesometrial decidua; cm, circular smooth muscle; c, conceptus; Endo, endometrium; ge, glandular epithelium; le, luminal epithelium; lm, longitudinal smooth muscle; Myo, myometrium; pdz, primary decidual zone.

On Day 4.5, just after the onset of implantation, *Figf* mRNA hybridization signals were localized (Figure 1A) to endometrial stromal cells of the endometrium of NIS tissue including glandular epithelial cells (Figure 1B–D) while signals in the myometrium were mainly in the connective tissue between the circular and longitudinal muscle layers of the myometrium (Figure 1E). In IS tissue, very strong *Figf* mRNA hybridization signals were seen in the primary decidual zone surrounding the implanting blastocyst (Figure 1F,G). Lower signals were also seen in the endometrial stromal cells and glandular epithelial cells of the endometrium (Figure 1H,I) as well as the connective tissue layer between the two layers of the myometrium (Figure 1J). Hybridization signals for Day 5.5 NIS (Figure 1K) were localized to similar regions as seen for the same tissue on Day 4.5 (Figure 1A) and 6.5 (data not shown). Although some hybridization signals were seen in the connective tissue between the layers of the myometrium, strong *Figf* mRNA hybridization signals were localized mainly to the primary decidual zone (PDZ) of the forming antimesometrial decidua of Day 5.5 (Figure 1L,M) and 6.5 (Figure 1N–P) IS tissues.

**Figure 2:**
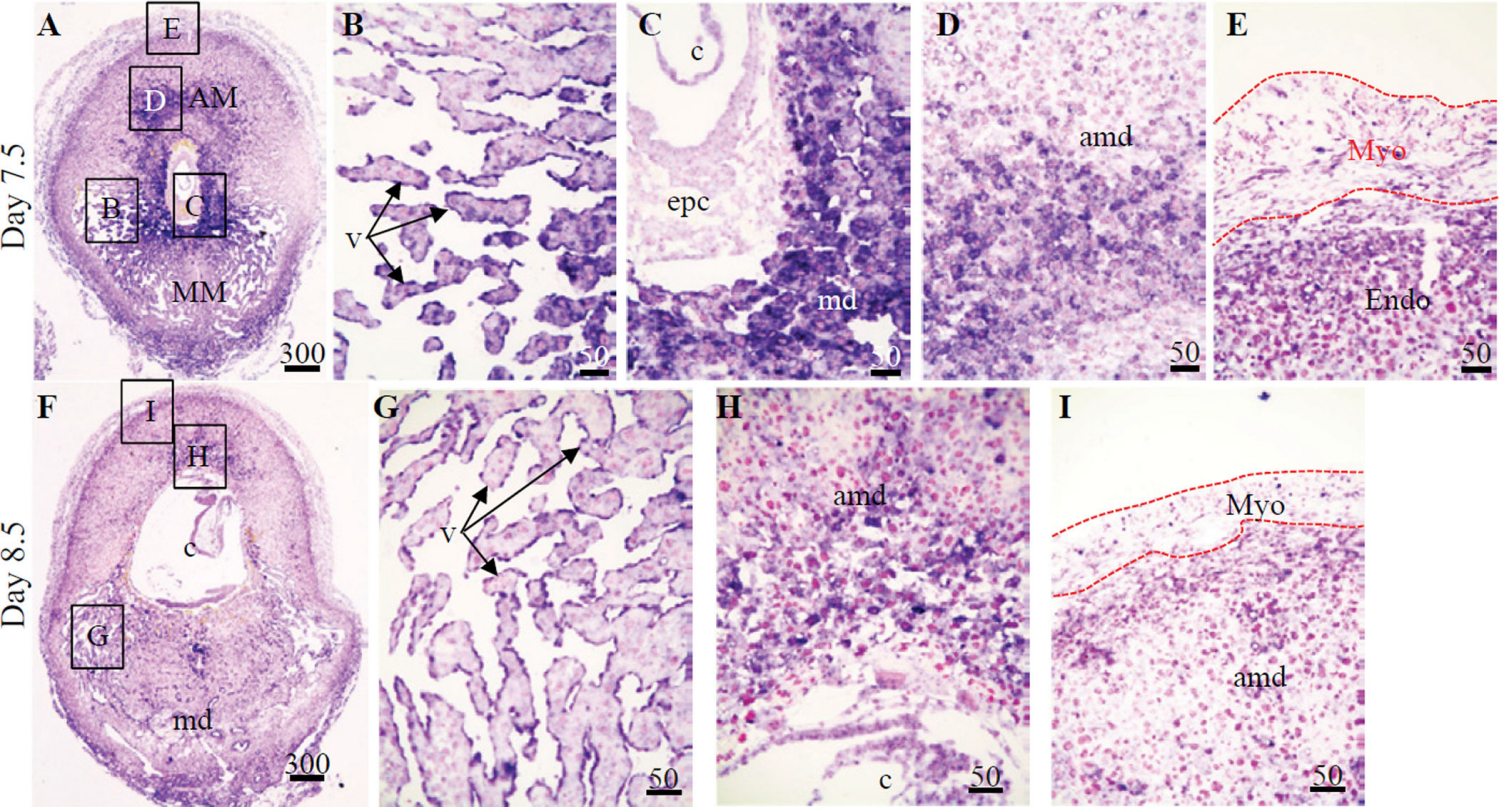
Localization of *Figf* mRNA in non-implantation (NIS) and implantation (IS) tissue segments from Day 7.5–8.5 pregnant mice. (**A–E**) Day 7.5 IS, (**F–I**) Day 8.5 IS. Global linear adjustments of the brightness and color level were made on the photomicrographs to more accurately represent what was seen on the slides under the microscope. Numbers above scale bars are in microns. Hybridization signal is purple while nuclear stain is pink. All sections in photomicrographs are oriented mesometrial (MM) and anti-mesometrial (AM) regions down and up, respectively. Abbrev: amd, antimesometrial decidua; c, conceptus; Endo, endometrium, epc, ectoplacental cone; md, mesometrial decidua; Myo, myometrium; v, vascular endothelial cells.

Hybridization signals for *Figf* mRNA in Day 7.5 IS tissues were strong in the endothelial cells of the forming sinusoid-like structures in the lateral mesometrial region, as well as in the antimesometrial plus mesometrial decidual cells surrounding the concept us (Figure 2A–D). Light hybridization signals were also seen in a thin layer of the endometrial stromal cells adjacent to the myometrium as well as in the connective tissue between the myometrial muscle layers (Figure 2A,E). By Day 8.5, strong hybridization signals remained in the endothelial cells of the sinusoid-like structures in the lateral mesometrial region of IS tissue (Figure 2F,G). However, noticeably lower signals were seen in antimesometrial plus mesometrial decidual cells surrounding the concept us (Figure 2F,H). Finally, light hybridization signals were also seen in a thin layer of the endometrial stromal cells adjacent to the myometrium as well as in the connective tissue between the myometrial muscle layers (Figure 2A,E) in Day 8.5 IS tissue (Figure 2I).

**Figure 3:**
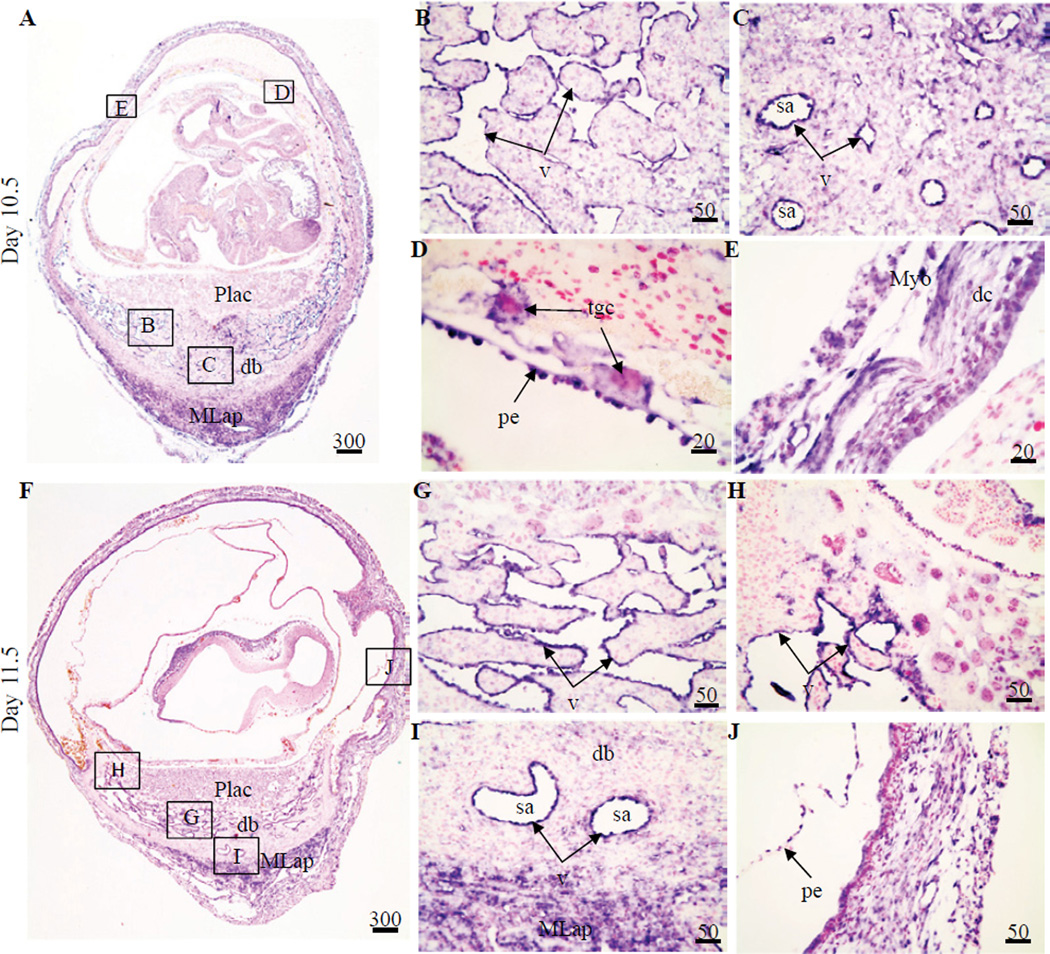
Localization of *Figf* mRNA in non-implantation (NIS) and implantation (IS) tissue segments from Day 10.5–11.5 pregnant mice. (**A–E**) Day 10.5 IS, (**F–J**) Day 11.5 IS. Global linear adjustments of the brightness and color level were made on the photomicrographs to more accurately represent what was seen on the slides under the microscope. Numbers above scale bars are in microns. Hybridization signal is purple while nuclear stain is pink. All sections in photomicrographs are oriented mesometrial and anti-mesometrial regions down and up, respectively. Abbrev: db, decidualbasalis; dc, decidua capsularis; MLap, mesometrial lymphoid aggregate of pregnancy; Myo, myometrium; Plac, placenta; pe, parietal endoderm; sa, spiral arteriole; tgc, trophoblast giant cell; v, vascular endothelium.

Once decidualization was complete on Days 10.5 and 11.5 IS tissue, strong *Figf* mRNA hybridization signals remained in the endothelial cells lining the sinusoids in the mesometrial decidua (Figure 3A,B,F–H). Most other endothelial cells of the mesometrial decidua also showed strong hybridization signals including the spiral arterioles (Figure 3C). Hybridization signals were found in the mesometrial lymphoid aggregate of pregnancy (MLap) (Figure 3A,F) as well as in yolk sac and some trophoblast giant cells of the concept us (Figure 3D,I). Low hybridization signals were also seen in the decidua capsularis and myometrium (Figure 3E,J). The localization of the signal in the myometrium appeared to include not only the connective tissue but also the myometrial cells (Figure 3E,J).

### *Figf* mRNA splice variant expression in the mouse uterus during implantation

**Figure 4:**
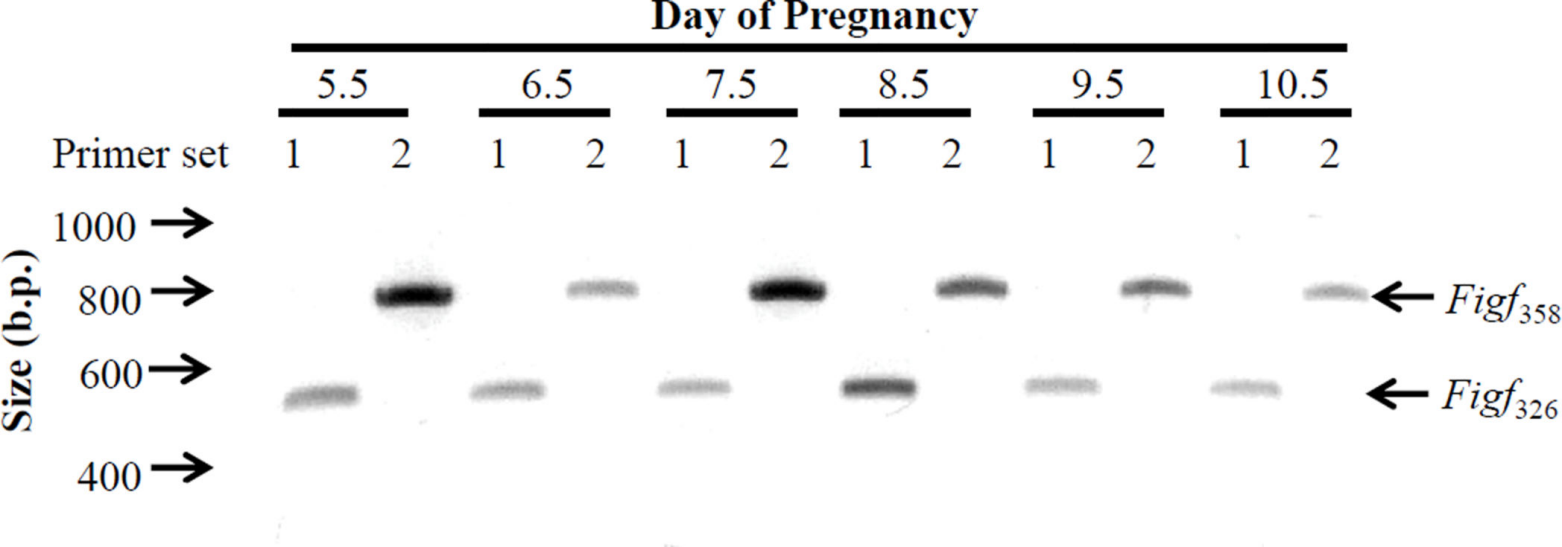
RT-PCR analysis of *Figf* mRNA splice variant expression in implantation site tissue segments from the uteri of Day 5.5–10.5 of pregnancy. Abbreviations: b.p., base pair; *Figf*_326_ (primer set 1) and *Figf*_358_ (primer set 2) amplicons are from the two mRNA splice variants which encode 326 and 358 amino acid FIGF proteins.

Two alternatively spliced mRNAs called *Figf*_326_ and *Figf*_358_,which encode 326 and 358 amino acid *FIGF* proteins, respectively, exist in the mouse. As shown in Figure 4, both alternatively spliced transcripts were detected by RT-PCR in IS tissues from Day 5.5–10.5 pregnant mice.

### *Flt4* mRNA localization in the uterus during implantation

**Figure 5:**
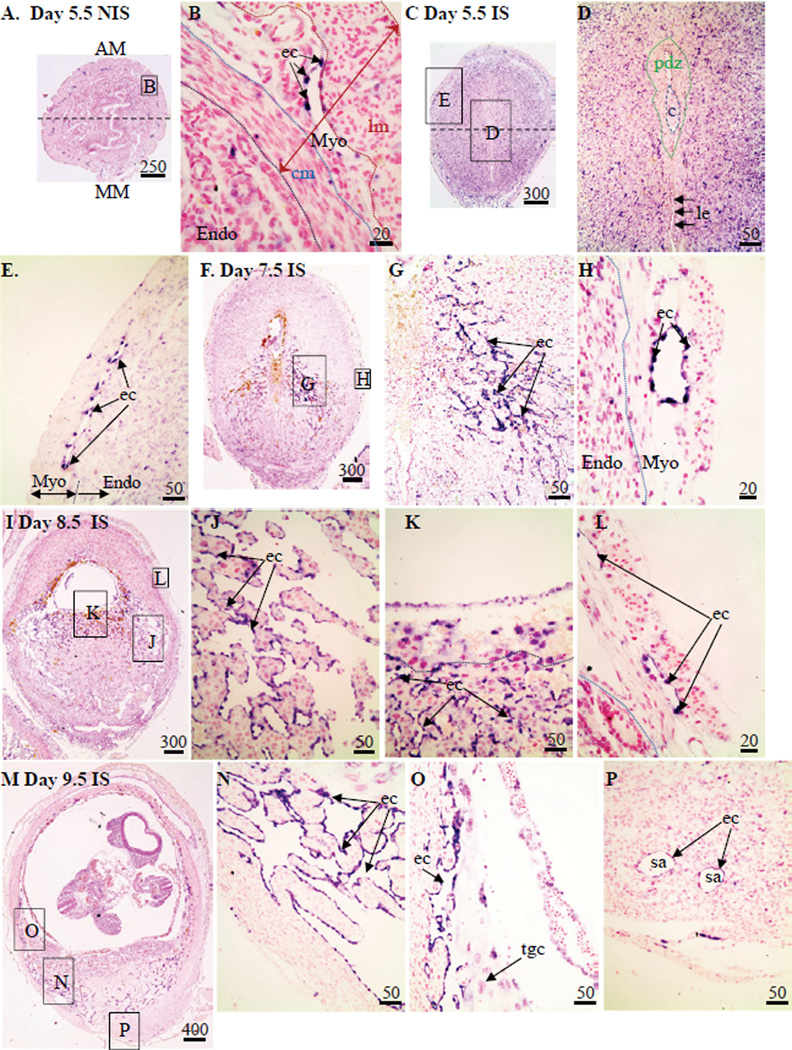
Localization of *Flt4* mRNA in non-implantation (NIS) and implantation (IS) tissue segments from Day 5.5–9.5 pregnant mice. (**A–B**) Day 4.5 NIS, (**C–E**) Day 4.5 IS, (**F–H**) Day 7.5 IS, (**I–L**) Day 8.5 IS and and (**M–P**) Day 9.5 IS. These are representative of at least 3 independent samples. Global linear adjustments of the brightness and color level were made on the photomicrographs to more accurately represent what was seen on the slides under the microscope. Numbers above scale bars are in microns. Hybridization signal is purple while nuclear stain is pink. All sections in photomicrographs are oriented mesometrial (MM) and anti-mesometrial (AM) regions down and up, respectively. Abbrev: c, conceptus; cm, circular smooth muscle; ec, endothelial cell; Endo, endometrium; lm, longitudinal smooth muscle; Myo, myometrium; pdz, primary decidual zone; sa, spiral arteriole; tgc, trophoblast giant cell.

*Flt4* mRNA was localized in the uteri of mice during implantation using in situ hybridization. *Flt4* mRNA hybridization signals were mainly localized to the endothelial cells in the connective tissue between the smooth muscle layers of the myometrium in the NIS tissues of Day 5.5 (Figure 5A,B) plus 6.5–9.5 (data not shown) pregnant mice. In addition to this connective tissue in the myometrium, hybridization signals for *Flt4* mRNA were seen in a subpopulation of cells scattered throughout the endometrium except for within the PDZ in Day 6.5 IS tissue (Figure 5C–E). By Day 7.5, 8.5, and 9.5, strong hybridization signals were seen in the endothelial cells lining the sinusoids in the lateral mesometrial decidua (Figure 5F,G,I–K,M–O), as well as in the endothelial cells in the connective tissue between the smooth muscle layers of the myometrium (Figure 5H,L) of IS tissue. By mid-pregnancy on Day 9.5, hybridization signals above background were seen in the endothelial cells of the spiral arterioles in the mesometrial region of IS tissue (Figure 5P).

### Proliferation of endothelial cells lining the mesometrial vascular sinusoids

**Figure 6:**
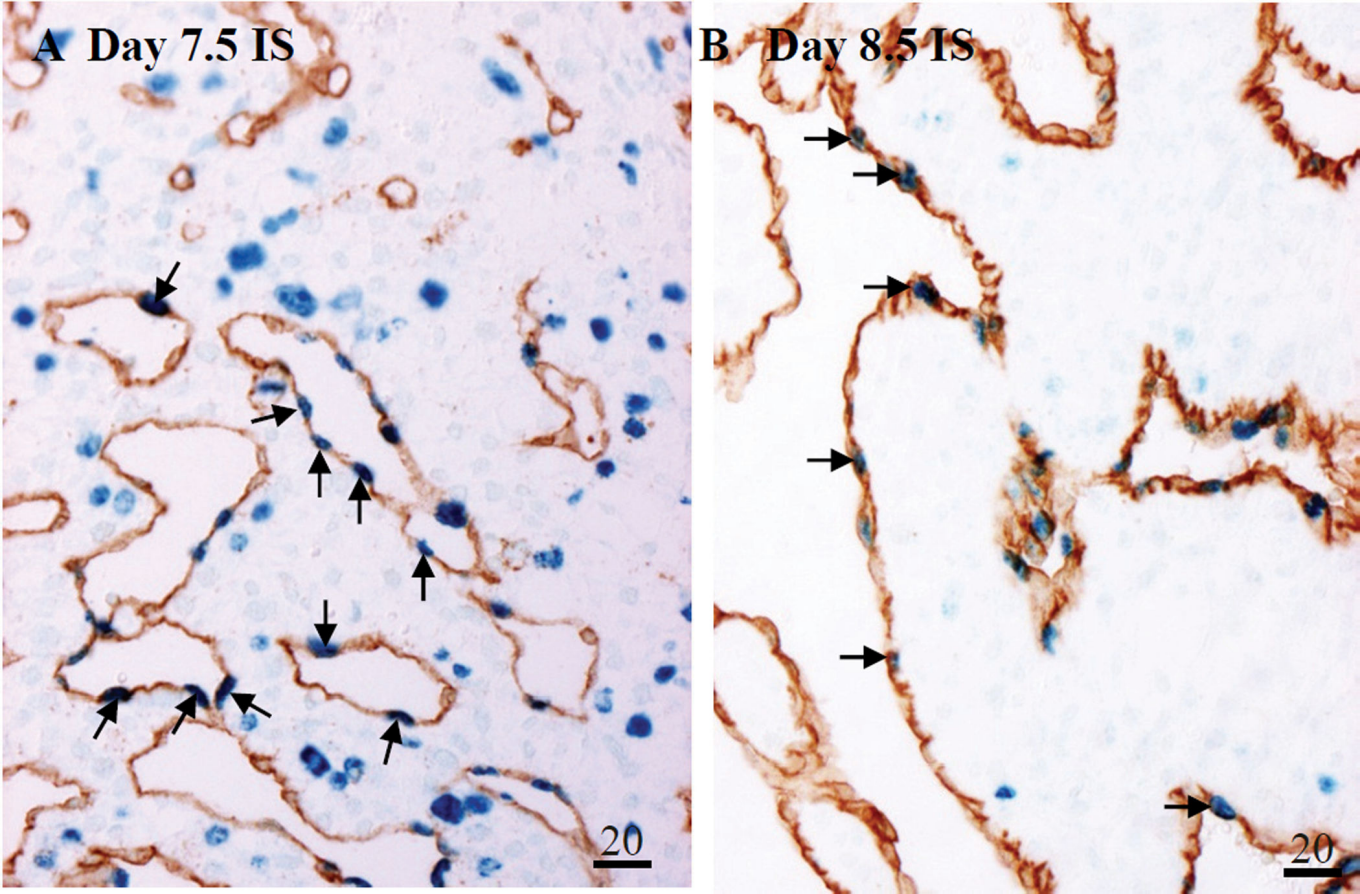
Localization of proliferating (BrdU^+^, blue) endothelial (Cd34^+^, brown) cells lining the lateral mesometrial sinusoids of Day (**A**) 7.5 and (**B**) 8.5 IS tissue. Numbers above scale bars are in microns. Global linear adjustments of the brightness were made on the photomicrographs to more accurately represent what was seen on the slides under the microscope. All sections in photomicrographs are oriented mesometrial (MM) and anti-mesometrial (AM) regions down and up, respectively. Arrows denote examples of BrdU^+^CD34^+^ proliferating endothelial cells.

Double immunohistochemistry was used to localize proliferating (BrdU-positive, BrdU+) endothelial (CD34-positive, CD34^+^) cells in Day 7.5 and 8.5 IS tissue. Since the endothelial sinusoids form during this period and highly express *Figf* mRNA we wanted to verify that the endothelial cells were proliferating, a sign of angiogenesis. Many BrdU^+^Cd34^+^ endothelial cells were seen lining the lumen of the vascular sinusoids in the lateral mesometrial decidua of Day 7.5 (Figure 6A) and 8.5 (Figure 6B) IS tissue.

## Discussion

The present study shows that *Figf* expression increases in IS tissue in a cell-type or region-specific fashion in the endometrium during implantation in mice. Although expression was seen in many cell types of the uterus in NIS tissue, there appeared to be a dramatic increase in *Figf* expression localized to the primary decidual zone, which forms just after the onset of implantation. By Day 6.5 of pregnancy, this heightened expression could be seen in a layer of decidual cells immediately surrounding the implanting concept us. On Day 7.5, this expression continued around the concept us but now there was also high expression of *Figf* in the endothelial cells that line the forming vascular sinusoids in the lateral mesometrial decidua. Interestingly, on Days 8.5 this high expression continued in the endothelial cells lining the sinusoids in the lateral mesometrial decidua cells but not the decidual cells surrounding the concept us. As implantation and placental development finish, heightened expression remained in the endothelial cells of the sinusoids and spiral arterioles of the decidua basalis. Noticeable heightened expression was also seen in the MLap. To our knowledge, this is the first detailed description of *Figf* mRNA expression in the mouse uterus throughout implantation.

Many forms of *FIGF* protein are produced by a combination of post-transcriptional alternative mRNA splicing and post-translational protein proteolysis plus glycosylation [20]. In the present study, we demonstrated that both of the known splice variants were expressed in the IS tissue of the mouse uterus during implantation. However, in order to effectively bind and activate receptors, there appears to be a requirement for posttranslational modifications of mouse and human *FIGF* [20,24]. Indeed, besides being essential for its function, the ability to process *FIGF* appears to be cell-specific [25]. Although there appears to be a conservation of the processing of mouse and human *FIGF* [20], it is generally thought that there is a major difference between mouse and human *FIGF* action. Human *FIGF* can bind to and activate KDR and *FLT4* [18,24], where as mouse *FIGF* appears to only bind to *FLT4* [19]. Therefore, it is likely that *FIGF* produced in the mouse uterus during implantation acts only on *FLT4* receptors. Presently it remains to be determined which isoforms of *FIGF* protein are found in the mouse uterus during implantation as well as which locally-produced proteolytic enzyme(s) in the uterus may be responsible for generating them.

*Figf* expression in the endometrium may be playing a role in formation of the vascular sinusoids in the lateral mesometrial decidua. Angiogenesis is the creation of new blood vessels from pre-existing ones and different types have been described [26–29]. However, the common feature of these different types of angiogenesis is endothelial cell proliferation. Similar to previous observations in the rat [30,31], the results of this study show that by Day 7.5 of pregnancy, there is intensive proliferation of the endothelial cells that line the sinusoids which form in the lateral mesometrial decidua. In the rat, suppression of uterine angiogenesis during this time prevents the formation of the sinusoids [32]. Although generally considered to mainly have a role in lymphangiogenesis, in some circumstances *FLT4* may also play a role in angiogenesis in adult tissues [1]. Since the present study demonstrates that the endothelial cells of the sinusoids express *Flt4* and a great deal of these cells are undergoing proliferation, we speculate that FIGF-*FLT4* signaling may be playing a role in endothelial angiogenesis in the mesometrial decidua. This speculation is supported by a recent finding where *Figf* overexpression in the uterus caused enlargement of endometrial (but not myometrial) blood vessels in mice [17]. Notably, however, previous results have shown *Kdr* and *Vegfa* expression in the endothelial cells lining the endometrial sinusoids and the decidua, respectively [33,34]. Therefore FIGF-*FLT4* signaling may be playing an overlapping role in endothelial angiogenesis during the formation of the sinusoids. Currently we need a better understanding of the molecular signaling involved in the formation of these sinusoids and their function as pregnancy progresses.

*FLT4* may influence the formation of a normal primary decidual zone. Recently, with the use of an antibody that blocks the functional activation of *FLT4*, it was suggested that *FLT4* is involved in angiogenesis of the primary decidual zone as evidenced by decreased PECAM1 staining [18]. However, because this study did not show *FLT4* localization to the primary decidual zone, an alternative explanation is that *FLT4* is indirectly involved in the survival of PECAM1-positive cells in the primary decidual zone. The present study did not localize *Flt4* mRNA to the primary decidual zone, supporting speculation that a direct action of *FIGF* is likely not on the primary decidual zone. Finally, although PECAM1 is found at high levels in the primary decidual zone [18,34], other markers of endothelial cells in the uterus such as KDR [34] and vWF [35] are not. Therefore, the suggestion that angiogenesis occurs in the primary decidual zone based on PECAM1 staining alone [18] may be premature. Even if blood vessels are present, little blood flow is reaching the primary decidual zone tissue in rodents [36–38]. Clearly more work is necessary to clarify if *FLT4* plays a role in formation of the primary decidual zone.

*Flt4* is expressed in the myometrium of the mouse uterus during implantation, where it is likely playing a role in lymphatic but not blood vessel remodeling. LYVE1 and prospero-related homeobox 1 (PROX1) proteins, markers for lymphatic endothelial cells, are mainly expressed in endothelial cells of the highly vascular connective tissue between the circular and longitudinal myometrial smooth muscle layers of the mouse uterus during implantation [39,17]. Previous work showed that these lymphatic endothelial cells of the myometrium stain positive for *FLT4* [18]. The present study also showed that the major site of *Flt4* and *Figf* mRNA production is located to the connective tissue between the muscle layers of the myometrium. Overall, this suggests that locally produced *FIGF* in the myometrium may regulate the activity of *FLT4*-positive lymphatic endothelial cells in the connective tissue between the muscle layers in the mouse during implantation. This speculation is strongly supported by a recent finding where *Figf* overexpression in the mouse uterus caused enlargement of myometrial lymphatic vessels in mice [17].

Although it is well-established that FLT1 and KDR have been shown to play roles in placental development and function [40–44], less is known about such possible functions for *FLT4*. Extravillous trophoblast cells of the human placenta stain positive for *FLT4* and *FIGF* proteins from 8–20 weeks [45]. Later in pregnancy, *FLT4* mRNA and protein as well as *FIGF* protein is present in in the term placenta of humans [46]. Finally abnormal villous development of the placenta in intrauterine growth-restricted pregnancies may involve abnormal *FLT4* signaling [47]. Therefore, limited human data suggests a function of *FLT4* signaling in human placental function. The present study localized *Figf* expression in trophoblast giant cells and other cells making up some of the extra-embryonic membranes of the placenta by mid-pregnancy. However, to the best of our knowledge, a role for *FIGF* in mouse placental development and function is currently not known.

In conclusion, the results of this study characterize the localization of *Figf* and *Flt4* mRNA expression in the mouse uterus throughout implantation in mice. This work will provide a basis for further research on the potential role of FIGF-*FLT4* signaling in endometrial angiogenesis during implantation in mice.
